# Comparison of *S. stercoralis* Serology Performed on Dried Blood Spots and on Conventional Serum Samples

**DOI:** 10.3389/fmicb.2016.01778

**Published:** 2016-11-08

**Authors:** Fabio Formenti, Dora Buonfrate, Rosanna Prandi, Monica Marquez, Cintia Caicedo, Eleonora Rizzi, Angel G. Guevara, Yosselin Vicuña, Francisco R. Huerlo, Francesca Perandin, Zeno Bisoffi, Mariella Anselmi

**Affiliations:** ^1^Centre for Tropical Diseases, Sacro Cuore HospitalVerona, Italy; ^2^Centro de Epidemiología Comunitaria y Medicina TropicalEsmeraldas, Ecuador; ^3^Instituto de Biomedicina, Carrera de Medicina, Universidad Central del EcuadorQuito, Ecuador; ^4^Laboratorio Clinico Centro de Salud Madre AnastasiaEsmeraldas, Ecuador

**Keywords:** *Strongyloides stercoralis*, dried blood spot, serology, prevalence, strongyloidiasis, diagnosis

## Abstract

**Background:** Dried blood spots (DBS) are used for epidemiological surveys on infectious diseases in settings where limited resources are available. In fact, DBS can help to overcome logistic difficulties for the collection, transport and storage of biological specimens.

**Objective:** To evaluate the accuracy of *Strongyloides stercoralis* serology performed on DBS.

**Methods:** A survey was proposed to children attending a school in the village of Borbon, Ecuador, and to their parents/guardians. Each participant gave consent to the collection of both serum and DBS specimens. DBS absorbed on filter papers were analyzed with a commercially available ELISA test for *S. stercoralis* antibodies, as well as with standard serology. The agreement between the two methods was assessed through the Cohen’s kappa coefficient.

**Results:** The study sample was composed of 174 children and 61 adults, for a total of 235 serum and 235 DBS samples. The serology was positive in 31/235 (13%) serum samples, and in 27/235 (11%) DBS: 4 samples resulted discordant (positive at standard serology). Cohen’s kappa coefficient was 0.921 (95% CI 0.845 – 0.998), indicating a high rate of concordance.

**Conclusion:** DBS are suitable for in field-surveys requiring serological testing for *S. stercoralis*.

## Introduction

Strongyloidiasis is the infection caused by the soil-transmitted helminth (STH) *Strongyloides stercoralis*. Its global prevalence has been largely underestimated until recent years, mostly because the diagnostic techniques commonly used for surveys conducted in the field on STH (such as Kato-katz technique, stool microscopy) are not appropriate for the detection of *S. stercoralis* (Bisoffi et al., 2013; Schar et al., 2013), hence they are not reliable to estimate its prevalence. Accurate estimates of prevalence are essential in endemic areas, to implement strategies for the control of this infection that, differently from the other STH, is potentially fatal in immunosuppressed individuals (Bisoffi et al., 2013; Krolewiecki et al., 2013; Buonfrate et al., 2015).

Among diagnostic tests for *S. stercoralis*, serology has demonstrated the highest sensitivity that makes it a good tool for screening and prevalence surveys (Krolewiecki et al., 2013; Bisoffi et al., 2014). Some Authors have concerns about its specificity (Siddiqui and Berk, 2001), particularly in settings where other STH are diffused, and could lead to cross-reactions. However, a previous study demonstrated that OD values above specific cutoffs reach virtually 100% specificity (Bisoffi et al., 2014). Common obstacles to the use of serology in surveys conducted in the field are due to storage (mostly lack of controlled systems that guarantee the maintenance of the samples within a given temperature range) and transport of the serum samples, particularly for studies in remote areas of the world. The collection of dried blood spots (DBS) on filter papers enables researchers to partially overcome these logistic problems. Moreover, fingerprick minimizes the discomfort of blood sampling for children and the biological risk for the phlebotomy staff (Sharma et al., 2014). Recently, Smit et al. (2014) reviewed the literature on studies that evaluated DBS assays compared to gold standards for the diagnosis of infectious diseases. In their analysis, the authors included 192 papers, mostly addressed to the diagnosis of HIV (24 studies). Among parasitic infections, malaria is the one with the highest number of studies (10), followed by lymphatic filariasis and one study on schistosomiasis (Downs et al., 2015). Globally, the use of blood samples collected on filter paper proved to be useful for the serodiagnosis of parasitic diseases, as well as for seroepidemiological surveys in human investigation. The use of DBS for epidemiological studies on strongyloidiasis has been evaluated only by two studies. Costa-Cruz et al. (1998) collected DBS from 207 individuals living in a rural area in Brazil, and analyzed the samples using an in-house immunofluorescence antibody test (IFAT). No comparison with a traditional method was performed, but the authors concluded that the results (7.7% positives) were reliable, according to the estimated prevalence in the study area.

A more recent paper by Mounsey et al. (2014) described the analysis of DBS using an ELISA test based on a recombinant antigen (NIE-ELISA). This study, conducted in an Aboriginal community of the Northern Territory of Australia, did not compare the results of the analysis on DBS to the results obtained with standard serology. However, on the basis of previous epidemiological studies, the authors concluded that DBS represent an adequate and useful tool for field studies.

Therefore, to the best of our knowledge no previous studies have directly compared the results of standard serology to the results of serology on DBS for the diagnosis of strongyloidiasis.

## Objective

The aim of this study was to evaluate the concordance between standard serology and serology performed on DBS for the screening of *S. stercoralis* infection.

## Methods

### Settings and Participants

**FIGURE 1:**
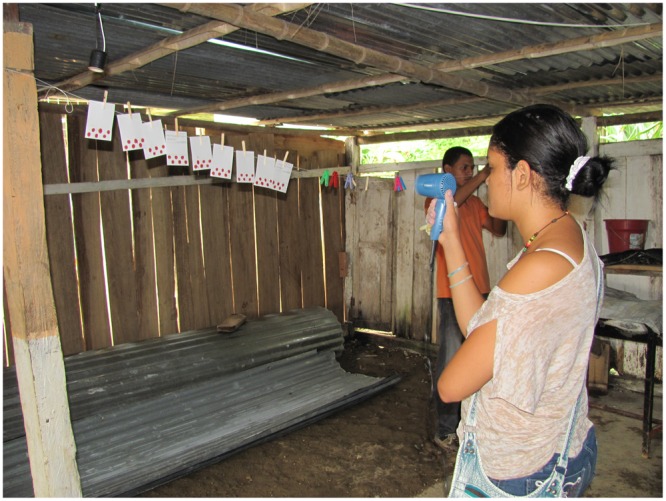
**Study settings.** Dried blood spots drying on filter papers.

A survey was conducted in the school “Unidad Educativa Mexico” of the village of Borbon, Ecuador, in December 2013. The survey was part of an extensive study for the evaluation of the impact of mass treatment with ivermectin (comparing both areas included and not included in the program), as described previously (Anselmi et al., 2015). Staff from the Centro de Epidemiología Comunitaria y Medicina Tropical (CECOMET) of Esmeraldas and of the Universidad Central del Ecuador, Quito, offered testing for *S. stercoralis* infection to all school children and to their parents or guardians. Blood specimens were obtained by venipuncture from each participant, and collected both in EDTA tubes and on filter papers (Whatman^®^ 3 mm, Maidstone, UK). All individuals accepted to collect a stool sample for stool microscopy, too. Filter papers were dried hanging on threads, with the aid of a hair dryer (**Figure 1**). Once dried, each filter paper was inserted in a plastic bag with silica gel. Five bags were then packed together in a second plastic bag, also containing silica gel. Eventually, those larger plastic bags were packed together in groups of five, with a further silica gel packet, in a third plastic bag, marked with the bio-hazard symbol.

The bags were stored locally at 4°C for no longer than a week, then transported to the Universidad Central del Ecuador, where they were kept frozen (-20°C) as it has been shown that IgG at -20°C remain stable for several years (Evengard et al., 1988; van den Akker et al., 1990; Behets et al., 1992). Finally, they were shipped to the Centre for Tropical Diseases (CTD) in Negrar, Verona, Italy, on January 2014 for analysis.

### Ethics

The study protocol was approved by the Ethics Committee of the Universidad Central del Ecuador (“Comité de Bioetica”— COBI) in November 2013 (IRB 00002438). Written informed consent was obtained from all participants (parents’ or guardians’ consent in case of children). The lab staff in Negrar carried out the analyses on fully anonymized, coded serum samples.

### Experimental Procedure

Serology was performed using a commercially available ELISA test (*Strongyloides ratti*, Bordier Affinity Products SA, Crissier, Switzerland) (Bisoffi et al., 2014). Sera in EDTA tubes were processed according to the manufacturer’s indications and to the routine testing procedures at CTD. The kit includes a weak positive control, which is used to assess the cutoff for positive results. As the cutoff varies between runs, we used a normalized Optical Density (OD) ratio (see section “Categorization of the results”) to compare the results obtained in different sessions.

Methods for testing the DBS followed three main steps, described hereafter.

Step 1. In consideration of adverse climate/humidity conditions at the moment of the collection of the DBS, and of the duration of transportation to Italy, a preliminary analysis was done in order to evaluate the integrity of the antibodies present in the samples, essential condition to proceed to the next steps. Hence, a biologist (FF) performed an electrophoresis on a polyacrilamide gel 4–18% on the eluated DBS samples of four individuals with stool microscopy positive for *S. stercoralis* (Supplementary Data Sheet 1). The elution was conducted overnight at room temperature in a buffer containing PBS, 0.05% tween 20 and protease inhibitor. The electrophoresis run for 3.5 h at 89 V. The bands corresponding to the IgG heavy (76 KDa) and light (26 KDa) chambers were evaluated to confirm the good preservation of the samples.
Step 2. Standardization of DBS processing methods. Several experiments were conducted by FF to evaluate the reproducibility of the results obtained from the eluted DBS samples, and the best method for the elution protocol. On the basis of the available literature, different temperature conditions and the presence/absence of a protease inhibitor were evaluated on eight DBS samples, comprising of four samples from “known” positive individuals and four presumptive negatives (on the basis of stool microscopy, Supplementary Data Sheet 1). Therefore, eight DBS were eluted overnight at room temperature in a buffer containing PBS, 0.05% Tween 20 (PBS-T) and preotease inhibitor and eight DBS samples were eluted in PBS containing 0.05% Tween 20 (PBS-T) overnight at 4°C without protease inhibitor. No differences were observed between these two conditions, which suggested that the use of inhibitors could be bypassed, performing the elution of DBS overnight at 4°C. Contrary to our expectation, no differences were observed comparing the elution at room temperature and at 4°C without protease inhibitor. With a prudent and conservative approach, we decided to perform the elution at 4°C.
The analysis was conducted in duplicates; the eluted samples were tested after dilution 1:50 and 1:201 (the latter being the one recommended by the manufacturer), in order to define the optimal dilution volume. The dilution 1:201 provided four positive and four negative samples (concordant with microscopy results), while at the dilution 1:50 all the eight samples were positive. On the basis of these results, the dilution 1:201 was adopted.
Step 3. A lab technician (ER), blinded to the results already obtained, performed the ELISA serology both on sera and on DBS, in parallel.

## Dbs Elisa Settings

Approximately 25 μl of blood were dropped on every circle of a diameter of 1.2 cm designed on filter paper. To extract a standard blood volume for the analysis, on the basis of the available literature (Parker and Cubitt, 1999), we used a 4.5 mm diameter punch. Considering the proportion of the blood volume contained in a 4.5 mm diameter punch, and an average haematocrit value of 40%, the elution of a single punch was performed in 421.2 μl of buffer. The ELISA test was then conducted as described above (“Experimental procedure” paragraph).

## Categorization of the Results

The tests were categorized according to the normalized OD (*OD* = absolute OD /cutoff ratio). In routine testing, the two categories are: positive (*OD* ≥ 1) and negative (<1). Based on the results of a previous study (Bisoffi et al., 2014), we also considered the category of “certain” cases, those with *OD* ≥ 2 (the test having been found to be virtually 100% specific over this OD). Borderline results (0.9 ≤*OD* ≤ 1.1) were re-tested and, if discordant, they were repeated by a different operator. The final result (positive/negative) was established considering two out of three concordant results.

## Statistical Methods

The agreement between the two alternative methods under study was assessed using Cohen’s Kappa coefficient. The values of Cohen’s Kappa coefficients were interpreted according to Landis and Koch: 1.00-0.81: excellent; 0.80-0.61: good; 0.60-0.41: moderate; 0.40-0.21: weak and 0.20-0.00: negligible agreement (Landis and Koch, 1977).

## Results

### Participants

Recruitment took place in December 2013. All individuals who accepted to participate were included. The total study sample was 235 people, comprising 174 children (age range 7–16) and 61 adults (age range 19–70). In total, 235 serum samples and 235 DBS were collected. Stool samples were also obtained from all participants.

### Test Results

**FIGURE 2:**
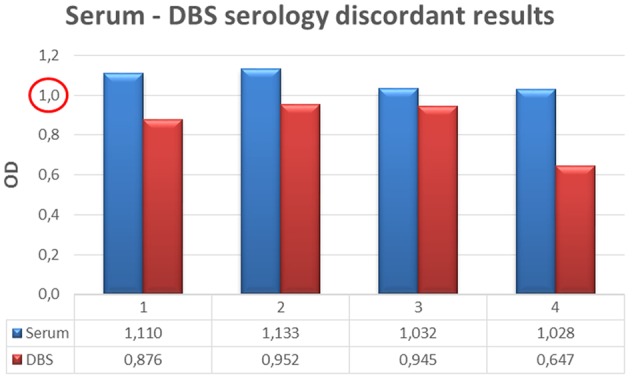
**Discordant results between DBS and standard serology considering OD cut off = 1**.

The serology was positive in 31/235 (13%) serum samples, and in 27/235 (11%) DBS (**Table 1**). Four of the 235 samples (2%) resulted discordant (serum positive, DBS negative). In particular, two of the four samples resulting negative in DBS were very close to the cutoff value (**Figure 2**). Overall, the Cohen’s Kappa resulted 0.921 (95% CI 0.845 – 0.998).

**Table 1:** Evaluation of the concordance of *S. stercoralis* ELISA performed on sera and on DBS.

	DBS +	DBS -	Totals
Sera +	27	4	31
Sera -	0	204	204
Totals	27	208	235

**K* = 0.921 (95% CI 0.845 – 0.998).*

Considering the OD cutoff value = 2 (as explained in Methods), nine serum samples and eight DBS resulted over cutoff. Three results were discordant (one *DBS* ≥ 2, serum < 2 and two serum ≥ 2, *DBS* < 2; **Table 2**). Cohen’s Kappa resulted 0.817 (95% CI 0.614 – 1.000).

**Table 2:** Evaluation of the concordance between standard serology and DBS, cutoff = 2.

	DBS +	DBS -	Totals
Sera +	7	2	9
Sera -	1	225	226
Totals	8	227	235

**K* = 0.817 (95% CI 0.614 – 1.000).*

All the results described so far were obtained considering a standard hematocrit (HCT) value of 40%. As the actual HCT value of the 174 children was known (mean value 36.8% ± 2.82 standard deviations), we were able to test the possible influence of the standard versus the actual value of HCT on the results on DBS. The results obtained by comparing the OD values of DBS considering a standard HCT value of 40% with those obtained considering each single HCT actual value are reported in **Table 3**. The concordance between the two methods remained high, with *K* = 0.917 (95% CI 0,824 – 1,000).

**Table 3:** Evaluation of the concordance between DBS considering an average HCT value of 40% and DBS considering each single HCT punctual value in children, cutoff = 1.

	DBS +	DBS -	Totals
Sera +	19	0	19
Sera -	3	152	155
Totals	22	152	174

**K* = 0.917 (95% CI 0.824 – 1.000).*

## Discussion

Prevalence figures of *S. stercoralis* have been largely based on fecal tests that lack sensitivity (Siddiqui and Berk, 2001; Knopp et al., 2008). Where serology has been used, prevalence figures were invariably higher (Loutfy et al., 2002; Buonfrate et al., 2015). Standard serology has limits as well, especially when performed in the field. Hence, the use of serology on DBS should represent an interesting diagnostic method for *S. stercoralis*, particularly for epidemiological studies when limited resources are available.

The results of this study showed a very good concordance between standard serology and DBS results: serology on filter papers demonstrated analogous performance to the “traditional” method; importantly, it did not cause a significant reduction in sensitivity. Moreover, the concordance was still good when considering *OD* > 2, that is the value virtually defining “certain positives.” Overall, it means that serology on DBS would give similar prevalence figures as traditional serology when used for serosurveys. Also, we demonstrated that there is no need for the precise, actual value of HCT to obtain a reliable calculation of the OD from the DBS. These results corroborate previous reports on the use of DBS for *S. stercoralis* (Costa-Cruz et al., 1998; Mounsey et al., 2014), although the previous studies lacked a direct comparison with standard serology and merely compared DBS results with previous estimates of prevalence. We believe that a main strength of this study is, precisely, the direct correlation of the results of conventional serology with those obtained on DBS. Moreover, for the first time serology for *S. stercoralis* was performed on DBS using a commercial ELISA kit, which does not require highly trained lab staff and is easily available.

Although serology may overestimate the prevalence of strongyloidiasis, its underestimation is potentially more harmful, leading to dangerously neglect the burden of this parasitic infection. Ideally, a combination of serology and appropriate fecal tests would provide the best estimation of *S. stercoralis* prevalence (Requena-Mendez et al., 2013). The results of the parasitological examination are reported as Supplementary Data Sheet 1, in order to give a complete figure of the work done.

Using blood samples from fingerprick, DBS serology is particularly suitable for field surveys and for use in children, thus allowing to obtain larger and possibly more reliable prevalence figures of *S. stercoralis* infection.

## Conclusion

There is a strong need for accurate prevalence data on *S. stercoralis* in many areas of the world, in order to implement adequate control strategies. We believe that DBS serology should play a role, being a reliable and feasible tool for field surveys even in particularly remote areas.

## Author Contributions

FF conducted the analysis and wrote the paper. DB contributed to write the paper. RP, MM, CC, and YV collected the samples on field. ER, performed the experiments. AG, FH, FP, and ZB revised the article. MA, planned the work and revised the article.

## Conflict of Interest Statement

The authors declare that the research was conducted in the absence of any commercial or financial relationships that could be construed as a potential conflict of interest.
